# Triglyceride to High-Density Lipoprotein Ratio can predict coronary artery calcification

**DOI:** 10.12669/pjms.38.3.5290

**Published:** 2022

**Authors:** Beilei Wang, Jinsheng Hua, Likun Ma

**Affiliations:** 1 Beilei Wang, Department of Cardiology, The First Affiliated Hospital of USTC, Division of Life Sciences and Medicine, University of Science and Technology of China, Hefei 230036, Anhui Province, P.R. China; 2 Jinsheng Hua, Department of Cardiology, The First Affiliated Hospital of USTC, Division of Life Sciences and Medicine, University of Science and Technology of China, Hefei 230036, Anhui Province, P.R. China; 3 Likun Ma, Department of Cardiology, The First Affiliated Hospital of USTC, Division of Life Sciences and Medicine, University of Science and Technology of China, Hefei 230036, Anhui Province, P.R. China

**Keywords:** Coronary artery disease, Coronary artery calcification, Triglycerides, High Density lipoprotein cholesterol, Risk factor, Prediction model

## Abstract

**Objectives:**

We assessed the TG/HDL-C ratio as a predictor for the presence of coronary artery calcifications (CACs).

**Methods:**

We collected demographic characteristics (age and gender), physical examination (height, weight, BMI, SBP, DBP), comorbidities, medication use, and laboratory variables Triglyceride to High-Density Lipoprotein (TG, HDL-C, TG/HDL-C, UA, TBG, 25-OH-VitD_3_); and we used coronary angiography to determine the presence of CACs. We performed univariate and multivariate analyses to evaluate the correlation between the TG/HDL-C ratio and CACs and established a predictive model.

**Results:**

CAC was present in 121 patients (25.80%). The levels of TG and TG/HDL-C ratio in the CAC group were higher than those in the non-CAC group, while the level of HDL-C in the CAC group was lower than that in the non-CAC group. The univariate analysis showed that the TG/HDL-C ratio was associated with CAC (OR, 0.021; 95% CI, 0.008 to 0.052; P<0.001), and the multivariate analysis indicated that the ratio was an independent risk factor for CAC (OR, 4.088; 95% CI, 2.787-5.996; P<0.001). Using the ratio to establish a prediction model, the area under the ROC curve was 0.814 (95% CI, 0.775-0.853; P<0.001), suggesting that the TG/HDL-C ratio has a high diagnostic efficiency. The diagnostic threshold was 1.037, and the corresponding sensitivity and specificity were 89.3% and 60.5%, respectively.

**Conclusion:**

The Triglyceride to High-Density Lipoprotein TG/HDL-C ratio is an independent risk factor for CAC with good diagnostic efficacy.

**Abbreviations:**
TG: Triglycerides
HDL-C: High-Density Lipoprotein
CAC: Coronary Artery Calcifications
BMI: Body Mass Index
SBP: Systolic Blood Pressure
DBP: Diastolic Blood Pressure
UA: Uric Acid
FBG: Fasting Blood Glucose
25-OH-VitD3: 25-Hydroxyvitamin D3
ACEI: Angiotensin-Converting Enzyme Inhibitors
ARB: Angiotensin Receptor Blockers
CCB: Calcium Channel Blockers
ARNI: Angiotensin Receptor-Neprilysin Inhibitor
CAG: Coronary Angiography
AUCROC: Area Under the Receiver Operating Curve

## INTRODUCTION

Vascular calcifications are mineral deposits on the walls of arteries and veins that can cause clinical complications such as vascular sclerosis, atherosclerotic plaque rupture, and heart failure.^1^,^2^ Coronary artery calcification (CAC) is thought to aggravate the prognosis of patients with coronary atherosclerosis. CAC is an important risk factor for the occurrence and progression of coronary heart disease and the occurrence of major cardiovascular events.^3^,^4^ Consequently, detecting artery calcification is important during the treatment of coronary heart disease.^5^ While many imaging strategies have been applied to CAC diagnosis, there are lack of serological markers that can be used to effectively predict CAC.

Triglycerides (TG) and high-density lipoprotein (HDL-C) are components of the atherosclerotic blood lipid profile^6^ that play important roles in the clinical assessment of the atherosclerotic heart disease risk.^7^ Studies have shown that the TG to HDL-C (TG/HDL-C) ratio can predict the risk and residual risk of coronary heart disease in the population, and it can give a comprehensive overview of an individual’s lipid metabolism with a higher clinical significance than looking at the TG and HDL-C levels separately.^8^ Although many studies have clarified the risk factors for CAC, no studies have assessed the association between the TG/HDL-C ratio and CAC. In this study, we evaluated the predictive value of the TG/HDL-C ratio for CAC.

## METHODS

We enrolled patients with suspected coronary heart disease hospitalized in the Department of Cardiovascular Medicine, at the Southern District of the First Affiliated Hospital of the University of Science and Technology of China from May 2020 to November 2020.

###  Inclusion criteria:

- Age between 18 and 80 years.
- CAG examination during a hospitalization with complete angiographic results in the medical records.
- Available relevant clinical data including demographic characteristics, past medical history, physical examination records, laboratory examination results, and others.

### Exclusion criteria:

- Patients with incomplete blood lipid profiles.
- Patients with combined severe liver and kidney damage, severe infection, rheumatic immune system diseases, or malignant tumors.
- Patients with diabetes and unsatisfactory blood sugar control.
- Malnourished patients.

Our research complied with the requirements of the 2013 edition of the Declaration of Helsinki. The ethics committee of the First Affiliated Hospital of USTC has approved this study (number: 2021-RE-069, date: 2021-July-02).

This is a retrospective case-control study, in which the relevant clinical data were obtained from the patient medical records and the electronic records system of the hospital. We extracted data including the patients’ age, gender, height, body weight, body mass index (BMI), systolic blood pressure (SBP), diastolic blood pressure (DBP); past medical history (recording the presence of hypertension, diabetes, or cerebrovascular disease); medication use (β-blockers, statins, angiotensin-converting enzyme inhibitors or angiotensin receptor blockers [ACEI/ARB], calcium channel blockers [CCB], metformin, or angiotensin receptor-neprilysin inhibitor [ARNI]); laboratory indicators such as HDL-C, TG, 25-hydroxyvitamin D3 (25-OH-VitD3), uric acid (UA), and fasting blood glucose (FBG) levels, and blood lipid-related derivative indicators such as the TG/HDL-C ratio. We collected the patients’ age, gender, past medical history, medication use status, height, weight, SBP, DBP, the waist circumference (measured on the admission day), the BMI (weight in kg/height in m^2^), the fasting blood (drawn the morning after admission), and the levels of HDL-C, TG, 25-OH-VitD3, UA, and FBG (tested in the hospital laboratory) from the clinical medical records. In addition, we calculated the TG/HDL-C ratio from the collected data.

### CAG and CAC diagnosis

All physicians performing CAG were cardiologist with cardiovascular intervention qualifications. The surgeons performed standard procedures for vascular puncture and left and right coronary angiographies, they identified CACs in high-density images with uneven density along the coronary vessels under X-rays, otherwise the procedures were labeled negative for CAC. Each CAC diagnosis was independently evaluated by two or more attending doctors from the cardiology interventional catheterization laboratory, and a third opinion was considered in cases of discrepancies.

### Statistical Analysis

We used R version 3.6.3 and EmpowerStats software to conduct the data analysis. Some of the graphs were drawn using Graphpad Prism 9. For variables with a few missing values in the data, we performed missing data analysis using multiple imputation methods with the EmpowerStats software. The measurement data was first tested for normality. We expressed normal distribution data as means ± SDs. We used independent sample t-tests for comparisons between the two groups. We expressed variables with non-normal distribution as medians (Q1, Q3). We used non-parametric U-tests for comparisons between groups. Enumeration data were expressed in the form of n (%), and the comparisons between groups were performed using chi-square tests. We applied multivariate logistic regression analysis to evaluate the independent role of the TG/HDL-C ratio in CAC, and expressed the results as odds ratios (ORs) and 95% confidence intervals (CIs). To establish a predictive model of the risk of developing CAC, we used the area under the receiver operating curve (ROC) (AUCROC), and we found the optimal threshold based on the Youden index. We considered all P values < 0.05 as statistically significant.

## RESULTS

We included data from 478 patients after following inclusion and exclusion screening criteria. The patients were divided into CAC (n=121 or 25.31%) and non-CAC (n=357 or 74.69%) groups based on the CAG results. The baseline information of the patients is shown in Table-I. TG/HDL-C values were organized in a descending order and the data was divided into two groups with the same number of members (one group with high values and one with low values). We performed univariate and multivariate logistic regression analyses of baseline indicators and TG/HDL-C values. The results are shown in Table-II. The univariate analysis suggests that the metrics associated with low TG/HDL-C levels are age (OR, 0.976; 95% CI, 0.959-0.994; P = 0.01), hypertension status (OR, 0.686; 95% CI, 0.477-0.987; P = 0.042), DBP elevation (OR, 0.981; 95% CI, 0.965 to 0.998; P = 0.027), UA (OR, 0.998; 95% CI, 0.996-0.999, P< 0.001), FBG (OR, 0.753; 95% CI, 0.646 to 0.877; P <0.001), and metformin levels (OR, 2.304; 95% CI, 1.248 to 3.313; P = 0.004). In addition, mixed cerebrovascular disease, elevated SBP, lowered 25-OH-VitD3, and lack of ACEI/ARB use tend to be associated with high TG/HDL-C levels, but did not show statistical significance. After including the above metrics in a multivariate analysis using the forward selection (conditional) method, only age (OR, 0.978; 95% CI, 0.976-0.980; P = 0.022), UA (OR, 0.998; 95% CI, 0.996 to 0.999; P = 0.002), FBG (OR, 0.779; 95% CI, 0.665-0.913; P = 0.002), and metformin (OR, 2.128; 95% CI, 1.283 to 3.528; P = 0.003) remained associated with the TG/HDL-C ratio.

**Table I:** Characteristics of the Population.

Clinical Features	Total(n=478)	Non-Calcification(n=357)	Calcification(n=121)	t/Z/χ^2^	P
Age (year)	63(55,68)	61(54,66)	68(62,73)	-6.875	<0.001
Sex (Male)	242(50.6)	163(45.7)	79(65.3)	13.933	<0.001
Smoking (Yes)	106(22.2)	75(21.0)	31(25.6)	1.114	0.291
Hypertension (Yes)	272(56.9)	191(53.5)	81(66.9)	6.657	0.010
Diabetes (Yes)	100(20.9)	55(15.4)	45(37.2)	25.922	<0.001
Cerebrovascular disease (Yes)	93(19.5)	58(16.2)	35(28.9)	9.271	0.002
BMI (kg/m2)	23.99(22.31,25.98)	23.96(22.48,25.82)	24.00(21.78,26.47)	-0.356	0.722
SBP(mmHg)	132.73±18.40	131.53±17.54	136.26±20.41	2.460	0.014
DBP (mmHg)	81.88±10.85	81.07±10.84	84.26±10.57	2.811	0.005
UA (μmol/L)	325.50(207.75,436.25)	315.00(200.50,411.00)	381.00(254.00,517.50)	-3.863	<0.001
FBG (mmol/L)	5.36(4.95,6.10)	5.30(4.97,5.69)	6.41(4.61,8.01)	-3.876	<0.001
25-OH-VITD (mg/L)	23.34(12.34,34.73)	24.12(12.32,37.42)	20.73(12.22,29.09)	-2.588	0.010
TG (mmol/L)	1.42(0.96,1.84)	1.25(0.82,1.72)	1.72(1.42,2.26)	-7.855	<0.001
HDL (mmol/L)	1.31(1.06,1.58)	1.38(1.17,1.64)	1.05(0.88,1.24)	-9.447	<0.001
TG/HDL	1.06(0.67,1.60)	0.92(0.55,1.29)	1.61(1.25,2.40)	-10.343	<0.001
β-blocker (Yes)	97(20.3)	70(19.6)	27(22.3)	0.409	0.522
Statin (Yes)	246(51.5)	194(54.3)	52(43.0)	4.674	0.031
ACEI/ARB (Yes)	156(32.6)	130(36.4)	26(21.5)	9.159	0.002
CCB (Yes)	142(29.7)	109(30.5)	33(27.3)	0.460	0498
Metormin (Yes)	84(17.6)	71(19.9)	13(10.7)	5.217	0.022
ARNI (Yes)	24(5.0)	19(5.3)	5(4.1)	0.268	0.604

**Table II:** Single factor and multivariate regression analysis of TG/HDL-C and baseline variables.

	Single factor analysis			Multi-factor analysis		
	
	OR (95% CI)	Wald	P value	OR (95% CI)	Wald	P value
Age (year)	0.976 (0.959~0.994)	6.573	0.010	0.978 (0.976~0.980)	5.270	0.022
Gender (Male)	0.791 (0.552~1.133)	1.639	0.201			
Smoking (Yes)	0.784 (0.509~1.209)	1.209	0.271			
Hypertension (Yes)	0.686 (0.477~0.987)	4.116	0.042			
Diabete (Yes)	0.701 (0.449~1.092)	2.465	0.116			
Cerebrovascular disease (Yes)	0.632 (0.400~1.001)	3.822	0.051			
Body Weight (kg)	1.000 (0.981~1.019)	0.002	0.961			
Height (m)	1.015 (0.992~1.039)	1.564	0.211			
BMI (kg/m2)	0.959 (0.898~1.026)	1.484	0.223			
SBP (mmHg)	0.991 (0.981~1.001)	3.196	0.074			
DBP (mmHg)	0.981 (0.965~0.998)	4.896	0.027			
UA (μmol/L)	0.998 (0.996~0.999)	13.604	<0.001	0.998 (0.996~0.999)	9.964	0.002
FBG (mmol/L)	0.753 (0.646~0.877)	13.301	<0.001	0.779 (0.665~0.913)	9.462	0.002
25-OH-VitD_3_ (mg/L)	1.012 (0.998~1.027)	2.905	0.088			
β-blocker (Yes)	0.752 (0.480~1.177)	1.560	0.212			
Statin (Yes)	1.034 (0.722~1.480)	0.034	0.855			
ACEI/ARB(Yes)	1.466 (0.997~2.153)	3.791	0.052			
CCB(Yes)	1.000 (0.675~1.480)	0.001	0.999			
Metformin (Yes)	2.034 (1.248~3.313)	8.120	0.004	2.128 (1.283~3.528)	8.545	0.003
ARNI (Yes)	0.702 (0.305~1.613)	0.696	0.404			

Mean TG level in the CAC group was 1.720 mmol/L (1.430 to 2.230), mean HDL-C level was 1.050 mmol/L (0.880 to 1.240), and mean TG/HDL-C level was 1.613 mmol/L (1.250 to 2.392). Mean TG level in the non-CAC group was 1.250 (0.820 to 1.710) mmol/L, that of the HDL-C level was 1.380 (1.170 to 1.640) mmol/L, and that of the TG/HDL-C level was 0.921 (0.556 to 1.288). Thus, the mean levels of TG and TG/HDL-C in the CAC group were higher than those in the non-CAC group (P<0.001), while the mean level of HDL-C was lower in the CAC group than in the non-CAC group (P<0.001).

We also conducted a univariate logistic regression analysis on patient’s demographic characteristics, past medical history, medication status, physical examination records, serological indicators, and atherosclerosis blood lipid profiles (Table-III). Univariate logistic regression results suggested three variables that may diminish the CAC risk: a high HDL-C value (OR, 0.021; 95% CI, 0.008 to 0.052; P<0.001), statin use (OR, 0.633; 95% CI, 0.418 to 0.960; P = 0.031) and metformin use (OR, 0.485; 95% CI, 0.258 to 0.912; P = 0.025). The risk factors for calcification in our analyses included age (OR =1.073; 95% CI, 1.048 to 1.09, P<0.001), male gender (OR, 2.239; 95% CI, 1.459 to 3.436, P<0.001), hypertension, (OR, 1.760; 95% CI, 1.142 to 2.711; P = 0.010), diabetes (OR, 3.251; 95% CI, 2.037 to 5.188, P<0.001), SBP (OR, 1.014; 95% CI, 1.003 to 1.026; P = 0.015), TG (OR, 2.592; 95% CI, 2.147 to 4.059, P<0.001), TG/HDL-C (OR, 4.024; 95% CI, 2.881 to 5.620, P<0.001), UA (OR, 1.003 ; 95% CI, 1.002 to 1.005, P<0.001), and FBG (OR, 1.716; 95% CI, 1.455 to 2.025, P<0.001). After applying a forward selection method (conditional) by including the TG/HDL-C ratio (excluding TG and HDL-C) into the multivariate logistic regression, our results shown that the TG/HDL-C ratio is an independent risk factor for CAC (OR =4.088; 95% CI, 2.787 to 5.996, P<0.001), while age (OR, 1.068; 95% CI, 1.038 to 1.099, P<0.001), male gender (OR, 2.558; 95% CI, 1.456 to 4.493; P = 0.001), diabetes (OR, 3.987; 95% CI, 2.145 to 7.410, P<0.001), cerebrovascular disease (OR, 1.946; 95% CI, 1.016 to 3.730; P = 0.045), UA (OR, 1.002; 95% CI, 1.000 to 1.004; P = 0.028), and FBG (OR, 1.530; 95% CI, 1.256 to 1.864, P<0.001) are statistically significant factors of the model (P<0.05).

**Table III:** Single factor and multi-factor regression analysis of calcification and baseline variables.

	Single factor analysis			Multi-factor analysis		

	OR (95% CI)	Wald	P value	OR (95% CI)	Wald	P value
Age (year)	1.073 (1.048~1.099)	33.979	<0.001	1.068(1.038~1.099)	20.014	<0.001
Gender (Male)	2.239 (1.459~3.436)	13.599	<0.001	2.558(1.456~4.493)	10.668	0.001
Smoking (Yes)	1.295 (0.801~2.095)	1.110	0.292			
Hypertension (Yes)	1.760 (1.142~2.711)	6.574	0.010			
Diabete (Yes)	3.251 (2.037~5.188)	24.442	<0.001	3.987(2.145~7.410)	19.117	<0.001
Cerebrovascular disease (Yes)	2.098 (1.294~3.402)	9.033	0.003	1.946(1.016~3.730)	4.025	0.045
Body Weight (kg)	0.989 (0.967~1.011)	1.020	0.312			
Height (m)	0.989 (0.963~1.016)	0.630	0.427			
BMI (kg/m2)	0.999 (0.926~1.078)	0.001	0.979			
SBP (mmHg)	1.014 (1.003~1.026)	5.923	0.015			
DBP (mmHg)	1.028 (0.989~1.048)	0.546	0.517			
UA (μmol/L)	2.952 (2.147~4.059)	44.361	<0.001			
HDL-C (mmol/L)	0.021 (0.008~0.052)	69.352	<0.001			
TG/HDL-C	4.024 (2.881~5.620)	66.724	<0.001	4.088(2.787~5.996)	51.886	<0.001
UA (μmol/L)	1.003 (1.002~1.005)	18.780	<0.001	1.002(1.000~1.004)	4.857	0.028
FBG (mmol/L)	1.716 (1.455~2.025)	41.122	<0.001	1.530(1.256~1.864)	17.852	<0.001
25-OH-VitD_3_ (mg/L)	0.976 (0.960~0.992)	8.103	0.010			
β-blocker (Yes)	1.178 (0.713~1.944)	0.409	0.523			
Statin (Yes)	0.633 (0.418~0.960)	4.639	0.031			
ACEI/ARB(Yes)	0.921 (0.874~1.047)	8.925	0.132			
CCB(Yes)	0.853 (0.539~1.350)	0.459	0.498			
Metformin (Yes)	0.485 (0.258~ 0.912)	5.050	0.025			
ARNI (Yes)	0.767 (0.280~2.100)	0.267	0.605			

**Fig.1:**
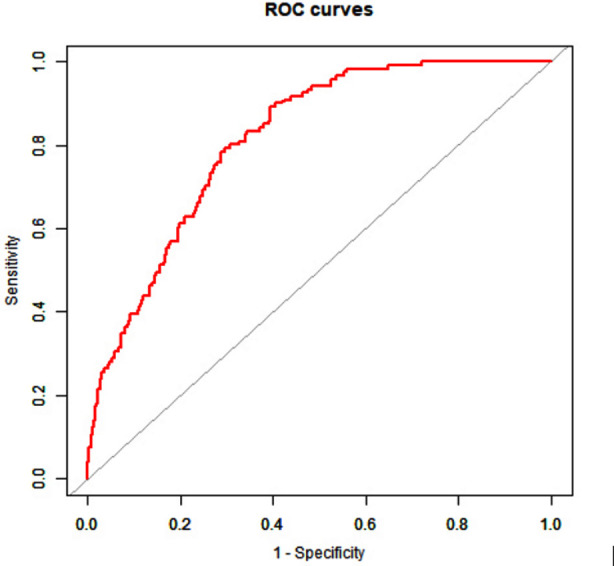
ROC curve of our TG/HDL-C prediction model.

Based on the results of the logistic regression analysis, we constructed a model using TG/HDL-C values to predict CAC, and calculated ROC curve. The regression equation is logit (P) = –3.00920 + 1.39223 x TG/HDL-C. As shown in Fig.1, the ROC AUC of TG/HDL-C values to predict calcification is 0.814 (0.775 to 0.853), corresponding to a P value of less than 0.05, and indicating that prediction based on the TG/HDL-C ratio is statistically significant. The threshold of the ratio to predict CAC is 1.037, the corresponding sensitivity is 89.3%, and the specificity is 60.5%.

## DISCUSSION

We found that TG/HDL-C ratios of patients differ significantly between CAC and non-calcification groups. Based on our multivariate logistic regression analysis, TG/HDL-C ratio is an independent risk factor for CAC (OR, 4.088; 95% CI, 2.787 to 5.996), suggesting that the ratio can be used as a new CAC biomarker. We also constructed a predictive model for CAC based on TG/HDL-C values, with an AUCROC of 0.814, (95% CI, 0.775 to 0.853) and a P<0.05, suggesting a satisfactory diagnostic performance. Based on these results, the TG/HDL-C ratio can effectively predict CAC in a clinical setting.

As the incidence of coronary heart disease is increasing over the years, the “early-diagnosis, early-treatment” strategy has become more and more important in the prevention of the life -threatening disease.^9^ Risk factors for vascular calcification include aging, male gender, diabetes, abnormal lipid metabolism, hypertension, smoking, abnormal renal function, inflammation, and others.^10^-^12^ Considerable progress has been made in the treatment of coronary heart disease, but its prevalence and morbidity remain high, and the mortality is still increasing. CAC, a form of coronary vascular disease, is also an important risk factor for coronary heart disease according to studies conducted in the field. Joseph et al.^13^ identified CAC and other factors (such as carotid artery intima-media thickness, ankle-brachial index, high-sensitivity C-reactive protein, and a family history of coronary heart disease) as cardiovascular disease risk factors on 6814 patients without cardiovascular disease. Follow-up studies indicated that CAC is an independent risk factor for coronary heart disease and cardiovascular disease, and that it has the highest predictive value of the evaluated risk factors. The research of Budoff MJ, et al. reached a similar conclusion.^14^ In addition, CAC in patients with coronary heart disease indicates a poor prognosis, and it induces calcified plaque formation, which becomes an independent risk factor for plaque rupture.^15^ Domestic scholars have confirmed that moderate to severe CAC in patients undergoing percutaneous coronary intervention is an independent risk factor for long-term major adverse cardiovascular events (HR=1.242; P = 0.017).^16^ However, few serological markers can effectively predict CAC.

The atherosclerotic blood lipid profile has remained a hot spot in coronary heart disease research. According to a large number of epidemiological studies, both high TG and low HDL-C levels are significantly associated with the risk of atherosclerotic cardiovascular disease, that is, an elevated TG is a risk factor, and a normal HDL-C level is a protective factor. The mechanism by which TG leads to coronary heart disease is unclear. However, the TG levels seem to regulate the levels of low-density lipoprotein and HDL-C. HDL-C reversely transports cholesterol from the blood vessel walls to the liver. The TG/HDL-C ratio responds rapidly to metabolism changes and is a sensitive indicator of the coronary heart condition.^17^

 Smooth muscle cells are important components of the muscular arterial wall and are involved in vascular calcification.^18^ Under pathological conditions, these cells change into secretory smooth muscle cells, macrophage-like or foam cells, osteogenic cells or chondrocytes.^19^ Animal experiments have found that inflammation plays an important role in the differentiation that turns smooth muscle cells into osteoblasts or chondrocytes.^20^ There is strong evidence that cardiovascular diseases are relevant to inflammation.^21^,^22^ Similarly, human clinical studies have confirmed that inflammation is an important risk factor for vascular calcification.^23^ Non-alcoholic fatty liver disease, an independent risk factor for CAC, is positively correlated with high levels of inflammatory indicators and is negatively correlated with adiponectin levels that confer anti-inflammatory effects. The TG/HDL-C ratio can also be used as a predictor of non-alcoholic fatty liver disease, suggesting that these factors are interrelated.^24^ In addition, studies have found a superoxide dismutase gene polymorphism associated with TG/HDL-C levels, which suggests that a high TG/HDL-C ratio indicates a high oxidative stress level,^25^ and that oxidative stress promotes cellular ossification of smooth muscle cells.^26^ This correlation also suggests an association between high TG/HDL-C values and CAC via oxidative stress.

We also identified age, male gender, diabetes, cerebrovascular disease, UA, and FBG as risk factors for CAC in patients. In agreement with these findings, other studies have shown that traditional cardiovascular risk factors such as advanced age, gender, diabetes, cerebrovascular disease, and blood sugar^27^ are influencing factors of CAC. Hyperuricemia is associated with metabolic syndrome;^28^ therefore, metabolic syndrome may mediate the correlation between hyperuricemia and CAC. In addition, we found an association between a high TG/HDL-C ratio and advanced age, high UA, and high FBG. Our results also suggest that the use of metformin is associated with a TG/HDL-C reduction. This adds to the evidence of the association between metabolic disorders and the TG/HDL-C ratio.

### Limitations of the study

Firstly, this study is based on single-center retrospective data, and the sample size was small. The data may be skewed by untested confounding factors in our analysis. Future studies should increase the study population size to obtain more representative results. Secondly, we assessed CAC simply by the presence or absence of calcifications, but we did not assess volume and density of the calcifications. Studies have found a positive correlation between the volume of a calcification and the risk of cardiovascular disease, and a negative correlation between the density and the same risk.^29^ Thus, our prediction of the risk of CAC and cardiovascular adverse events based on the TG/HDL-C ratio needs to be confirmed in detailed grouping, large sample size, and prospective studies. We are aware that other components of the atherosclerotic lipid profile, such as total cholesterol, low-density lipoprotein, and related derivative indicators, can be also important predictors of CAC.

## CONCLUSION

We found that the TG/HDL-C ratio in the blood lipid profile of atherosclerosis can effectively predict CAC. We believe our findings are of clinical importance because the TG/HDL-C ratio is a simple and easy-to-obtain peripheral blood marker.

### Authors’ contributions:

**BW** conceived and designed the study.

**JH and LM** collected the data and performed the analysis.

**BW** was involved in the writing of the manuscript and is responsible for the integrity of the study.

**LM** Analysis of data and edited the manuscript.

All authors have read and approved the final manuscript.
